# Supplementary material for: Are carbon emissions trading and green financial instruments synergistic? -Comprehensive quantitative research based on content analysis
Source: PLoS One. 2024 Mar 7;19(3):e0298601. doi: 10.1371/journal.pone.0298601 (PMC10919601; doi:10.1371/journal.pone.0298601)
Supplement: S1 Data — (ZIP) [file pone.0298601.s001.zip › Data/地级市Z_F工作报告.zip!/┴╔─■/╕o╦│╩╨/└┤╫╘╣1⁄2╓┌║┼í╛┬φ┐╦╩2╛▌═°í┐_2019.docx]

各位代表：

　　现在，我代表市政府，向大会报告工作，请予审议，并请市政协委员提出意见。

　　一、2018年工作回顾

　　2018年是本届政府的开局之年，也是抚顺振兴发展史上极不平凡的一年。一年来，我们坚持以习近平新时代中国特色社会主义思想为指导，在省委、省政府和市委的坚强领导下，在市人大和市政协的监督支持下，认真践行“为民多办细事、为城多解难事、为政多干实事”，团结带领全市人民“重实干、强执行、抓落实”，担当作为，埋头苦干，砥砺奋进，经济发展由企稳回升迈向总体向好，社会事业由重点突破转向全面进步，矛盾问题由叠加交织走向大幅递减，为振兴发展打下坚实基础。

　　——一批重大支撑项目快速推进、拉动强劲。清原抽水蓄能电站项目加快建设，沈白客专项目完成可研规划，总投资195亿元恒大康颐生态和养生谷两个项目正式启动，投资50亿元抚顺国际物流商贸城项目开工建设，抚顺铝业35万吨阳极炭素等项目建成投产，辽中环高速抚顺段竣工通车。

　　——一批重点产业平台加快建设、支撑有力。与沈阳经济区、沈抚新区深度融合发展，经济开发区与沈抚新区共建共享，望花开发区晋升省级开发区并获批省自贸区协同发展产业园区，东洲区获批全国第二批重点采煤沉陷区综合治理试点，高新区列入2018国家开发区公告审核目录。

　　——一批惠民利企新政集中出台、活力迸发。创新制定“飞地经济”“一牌两卡”等11项政策，减免企业税费10.6亿元、减少电费支出1.1亿元，新增市场主体2.1万户，其中新增企业4460户，实现“个转企”706户、“小升规”31户、“限下转限上”34户，创业信心、发展活力显著增强。

　　——一批先行先试举措高效落实、优势凸显。争取到建设项目环评审批等3项省自贸区政策，全面落实25项省自贸区改革创新经验，五大国有平台公司投融资26.5亿元、撬动项目18个、惠及企业70户，东洲区保税物流中心项目进入申报审批，人力资源产业园建成运行，冰雪、健康等新产业加快培育、竞相发展。

　　——一批历史遗留问题有效破解、负担锐减。13个历史遗留问题解决销号10个，解决“办证难”“回迁难”3.4万户、超额完成年度计划，制约发展的临江路东延工程竣工通车、雷锋路西延工程主体完工。不遗余力推动采煤影响区综合治理，导入产业项目28个，开创性整理土地4955亩，其中复垦耕地1200亩。特别是多渠道、不间断攻关争取，以西露天矿为核心的采煤影响区综合治理工作，得到了党中央高度重视、上升为国家研究战略，找到了破解重大历史难题的出路。

　　困境中前行，逆境中发展，我们有决心、有力量、有成果。特别是去年9月28日，习近平总书记亲临抚顺考察并作出重要指示，为我们指明了前进方向，给予了巨大关怀激励。这是我市振兴发展史上具有里程碑意义的大事，是我们抚顺人夙兴夜寐、奋发图强赢得的机遇，全市上下无比自豪、无比振奋，加快发展的信心干劲空前高涨。

　　一年来，我们主要做了以下工作。

　　1.狠抓稳定增长。始终把稳增长作为压倒一切的任务，多措并举破难题、抓运行、扩总量，经济发展逆势而上、总体向好。全年预计实现地区生产总值1038亿元、增长3.2％，首次突破千亿元大关；规上工业增加值和总产值分别实现322.6亿元和1130亿元、增长6.3％和16.8％；全社会固定资产投资194亿元、增长1％；域外引资到位额131.4亿元、增长15％；一般公共预算收入（含新区）97.2亿元、增长10.3％；社会消费品零售总额648亿元、增长3％；新增城镇就业2.2万人，物价保持总体稳定，城乡居民人均可支配收入分别达32600元和14300元、增长7.5％和7％，均高于经济增速。

　　2.狠抓产业升级。紧扣工业立市、壮大实体经济，全力改造升级传统产业，完成重点技改项目105个、投资32亿元，转化重点科技成果60项，规上工业企业平均发明专利居全省第二，石化公司新产品增效25亿元，抚矿集团与德国西门子成功开展技术合作，抚顺特钢开发新品种176个，华丰公司等尖端产品广泛应用国防和国家重大工程，永茂建机等18个产品获评省名牌产品、创历史新高，8个特色名优品牌实现产值111亿元，传统产业重现生机活力。全力培育新兴产业，清华启迪等25家前沿科技企业注册我市，氢燃料电池、环保新材料等项目成功引进；恒益石化、特力环保等项目开工建设，聚丙烯蜡等6个项目进入中试；以大恒化工等企业为主的健康产业集群加快形成，清原县成为省中医药健康产业先导区；冰雪体育、冰雪旅游项目加快建设，开启四季旅游新模式，实现旅游总收入328亿元、增长15％，新产业新业态多元发展、成为新的增长点。

　　3.狠抓改革开放。坚持向改革开放要动力、添活力，深入推进供给侧结构性改革，国有企业“三供一业”全部分离移交，抚顺特钢债务重组基本完成，厂办大集体改革迎来突破，事业单位、党政群机构改革有序推进。持续深化金融体制改革，天和环保等3家企业在辽股交挂牌，金融小镇入驻企业51家，全市存贷比达52％，金融业增加值占地区生产总值7.1％，金融撬动更加有力。全力打造一流营商环境，“3450”改革提前完成，“证照分离”改革全面推开，取消各类证明253项，175个政务事项实现“最多跑一次”，政务事项集中进驻率居全省第一，市场活力充分释放，民营企业总数增长15％，从业人员占职工总数的85％。全方位包保企业，创新实行“项目管家”等制度，解决涉企困难问题350余个，“纠四风”“办事难”专项整治解决问题70余个，乱收费、乱罚款得到有效治理。持续放大“魅力中国城”效应，北京、上海、深圳等异地商会快速成立，全年招商签约项目81个、总投资512亿元，引进高端紧缺人才百余名，高效的服务、一流的环境，为我市赢得了声誉、赢来了各方投资。

　　4.狠抓区域振兴。始终坚持城乡融合发展，投入4200万元发展集体经济，发放“惠农贷”3.1亿元，新增农业龙头企业12家、省级示范家庭农场11家、省级示范合作社9家、产业经济发展试点村22个，土地承包确权登记完成98％，流转土地总面积35.37万亩，村集体经济稳步发展、态势良好，乡村振兴步伐加快。县乡财政体制改革走在全省前列，金库设立实现全覆盖，创建“一村一品”示范村20个、“一乡一业”示范乡镇3个，峡河乡等3个乡镇获评辽宁特产之乡，高湾冰雪体育小镇、救兵镇列为省级特色镇，财政收入2000万元以上乡镇12个、5000万元以上6个，石文镇、前甸镇达亿元规模，乡镇发展提质升级。8个工业产业园区产值900亿元、4个农产品加工集聚区收入12.36亿元、8个服务业集聚区收入460亿元、17个特色产业小镇产值17.54亿元、8个特色小城镇产值46.1亿元，东洲区、新抚区、清原县、望花区预计经济增长高于全市、全省增速，县区实力普遍增强。

　　5.狠抓三大攻坚。坚决提高政治站位，积极克服财政收支矛盾，全力守住工作底线，竭尽全力确保了43.9万离退休人员养老金按时足额发放，顺利推进机关事业单位养老保险并轨，提前完成61.6亿元政府债务化解任务，金融秩序稳定、风险可控。深入实施“十大脱贫巩固提升工程”，建立产业扶贫基地441个，重病贫困户全部免费治疗，1053户贫困户D级危房完成改造，2141名贫困人口实现脱贫、20个贫困村销号。坚决整改环保督察反馈问题，大伙房一、二级保护区工业企业和养殖场全部关闭，水质稳定在Ⅱ类标准。大力推行“河长制”“警长制”，治理流域面积200平方公里以上河流12条（段），22条（段）城市黑臭水体治理通过国家验收。植树造林22.1万亩，修复环城林带1.2万亩，松材线虫病防治取得阶段性成果。10吨以下燃煤锅炉全部拆除改造，城区空气质量达标天数279天，清原县、新宾县获评全国“百佳深呼吸小城”，浑河水利风景区成功晋升国家级，优美生态成为城市新名片。

　　6.狠抓民生保障。坚持以人民为中心，大力发展社会事业，高龄补贴等十大民生实事有效办结、兑现承诺。普惠幼儿园覆盖率超过省定目标，中小学课后服务试点启动，教育育人质量进一步提升。紧密型医联体建设扎实推进，药品加成全面取消，降低医疗费用1.44亿元。深入推进“春雨惠民”行动，煤精琥珀雕刻等入选国家传统工艺振兴目录。市民15分钟运动圈基本建成，第13届省运会我市勇夺第4名佳绩。养老服务质量有效提升，发放助学金3045万元，救助贫困妇女儿童4.7万人次，城乡低保、农村特困人员、孤儿救助、优抚对象供养标准分别提高5％、11％、36.5％、12％，困难群体生活得到可靠保障。大力推进城乡建设，创新开展智慧交通建设，改造干线公路147公里，城东公交停车场一期工程建成投用，新建改造农村公路593公里、“一事一议”村路474公里，城乡电网改造投入6.4亿元。改造棚户区1960户、老旧小区10个，完成“暖房子”工程17万平方米、惠及群众3400余户。城市供水二次加压泵站改造全面完成，4.9万农村人口饮水难问题如期解决，改造农村无害化厕所5000座，57％的乡镇实现垃圾分类处置。大力创新社会治理，“五全齐动”成效明显，巡逻警力增长41％，刑事案件发案量下降11％、为十年最低，“扫黑除恶”破获案件百余起，圆满完成重大安保任务。非洲猪瘟疫情有效防控。超额完成省信访“减存控增”任务，进京访登记案次、非访人次均下降40％。严格落实“三个责任”，安全生产事故大幅下降，我们抚顺人生活的更舒心、更富足、更有尊严。

　　7.狠抓效能提升。始终坚持市委有决策、政府有落实，群众有呼声、政府有回应，代表委员有建议、政府有行动。强力开展“重强抓”专项行动，全力推动各级政府和广大干部聚焦主责主业，咬定目标勇担当、扑下身子抓落实、铆劲攻坚破难题，用苦干实干巧干打开工作局面。进一步健全了任务下摆、专业督导、定期调度、审计监察、考评奖惩“五位一体”闭环工作机制，形成了市级领导以上率下、部门县区合力推进、乡镇街道一线作战的落实体系，心想群众、眼盯项目、狠抓落实的氛围浓厚，193项“重强抓”专项任务高效完成。大力推行依法文明行政，清理废除地方性法规、规章和规范性文件16份，重拳整治庸政懒政怠政，从严查处侵民害民行为，人大代表建议、政协委员提案解决率分别达83.9％和86.7％，均创历史新高，政府公信力、群众满意度进一步攀升。

　　各位代表，艰难困苦，玉汝于成。过去的一年，我们夜以继日、苦干实干，用朴实的劳动、辛勤的汗水，换来了各项事业新进步、赢得了重大机遇，成绩来之不易。这是省委、省政府和市委坚强领导的结果，是市人大和市政协监督支持的结果，更是全市人民团结奋斗的结果。在此，我代表市政府，向全市各族人民，人大代表、政协委员，各民主党派、工商联、无党派人士、人民团体、离退休老同志；向驻抚部队、武警官兵、公安干警，中省直和机关企事业单位；向广大企业家、创新创业者，以及所有关心、支持抚顺振兴发展的朋友们，表示崇高的敬意和衷心的感谢！

　　发展永无止境，振兴任重道远。对照“四个短板”“七破七立”，对标先进榜样、群众期盼，我们的工作还有很多差距：思想不够解放，“等靠要”的问题没有彻底消除，服务群众和企业效果有限；体制机制不活，项目落地难、办事难问题还没有根本解决；产业结构单一，项目储备少，新产业引进不足，固定资产投资波动较大、没有达到预期；对外开放力度不强，外贸出口低位运行，出口拉动贡献较小。特别是受财力等因素影响，民生投入有限，群众合理诉求没能逐一解决到位。对此，我们深深自责、时刻警醒，绝不视而不见、回避推卸，无论问题多复杂、多久远，解决难度有多大，都将一个一个化解、一件一件兑现，切实让我们的城市充满温度、充满希望，成为人人向往的新抚顺。

　　二、2019年重点工作

　　2019年，是决胜全面建成小康社会的攻坚之年，也是振兴发展必须率先作为的关键之年。今天的抚顺，现实挑战和重大机遇并存。国际经贸摩擦仍在继续、全球增长低于预期，国内经济下行压力增大、实体经济发展难度增加，我市财政收支矛盾突出、部分企业运行困难，稳增长、保民生、保运转考验重重。但是，历经拼搏奋斗，目前发展基础坚实、人人思进、众志成城，党政群改革改出了新气象、新面貌，全市上下比任何时候都更加向往振兴、更加务实求实。特别是习近平总书记亲临考察、把脉定向，让我们迎来了前所未有的重大机遇，鼓足了前所未有的信心勇气，集聚了前所未有的干劲力量。只要我们抢抓机遇敢亮剑、直面挑战勇闯关、埋头苦干真落实，就一定能够迎难而上、率先作为，谱写新时代振兴发展新篇章。

　　今年政府工作的指导思想是：以习近平新时代中国特色社会主义思想为指导，深入学习贯彻习近平总书记在抚考察和深入推进东北振兴座谈会上的重要讲话精神，牢牢把握“稳中求进”工作总基调，全面落实新发展理念和高质量发展要求，聚焦“四个短板”和“六项重点工作”，突出“三个深度融合”“四个抚顺”建设，坚持工业立市、工业强市、产业兴市，坚持“实、好、干”工作理念，以全面振兴全方位振兴为总目标，以采煤影响区综合治理与开发利用为有利契机，以振兴产业、培育新动能为主攻方向，以狠抓项目、扩大开放为核心根本，以服务企业、优化环境为关键任务，以改善民生、绿色发展为工作底线，牢记使命，解放思想，抢抓机遇，苦干实干，率先走出资源枯竭型城市转型新路，确保总书记重要讲话精神落地生根、开花结果。

　　今年经济社会发展预期目标是：全力实现“一个不低于、四个两位数”目标，地区综合实力提档晋位。地区生产总值增长不低于全省平均水平，规模以上工业增加值增长10％，全社会固定资产投资增长10％，一般公共预算收入增长10％，域外引资实际到位额增长10％，社会消费品零售总额增长7％，城乡居民人均可支配收入与经济增长同步。万元地区生产总值能耗下降3.4％，煤炭消费总量控制在1108万吨。尽管实现这些目标异常艰难，但为政一方、实现小康，我们必须自加压力、奋力一搏、造福一方。

　　1.聚焦城市转型，推动重点区域率先突破。加快资源枯竭型城市转型，是党中央赋予的重大政治任务，是决定我市命运的关键一步，必须坚决贯彻落实重要指示精神，全力打开资源枯竭型城市转型突破口。

　　强力推进采煤影响区综合治理与开发利用。坚持内生动力与借力发力并重并举，以强烈的使命担当，配合国家部委、智囊机构等，高效开展以西露天矿为核心的采煤影响区综合利用科学论证，推动方案早通过、项目早建设，尽快把亲切关怀、重大机遇转化为实际成果。充分学习借鉴徐州经验，举全市之力治理灾害、修复生态、导入产业，综合复绿850亩，复垦耕地1700亩，煤矸石利用等项目启动建设，金融小镇入驻企业100家以上，加快打造“绿树环绕、生态优美、产业兴旺、宜商宜游”的新区，使长期制约发展的“城市伤疤”成为城市新景观、引领发展主引擎，真正造福全市人民、惠及子孙后代。

　　深度融入沈阳经济区、沈抚新区发展。顺应区域融合联动发展的大势，充分利用沈阳经济区、沈抚新区和省自贸区政策叠加效应，以经济开发区、望花开发区为先导，大力承接沈阳汽车、飞机等产业外延外移，全力推动与华晨宝马等大企业的协作配套，共建“飞地”产业基地，引进重点产业项目10个以上、投资30亿元。深入推进与沈抚新区的融合互补发展，全力推进交通等互联互通，加快恒大康颐生态和养生谷等项目建设，共建重点项目13个。着力放大沈抚两市千万级人口优势，共建三京旅游大市场，大力推出精品旅游路线，全力吸引来抚旅游消费，把我市建成沈阳产业外移的承接地、休闲游览的后花园，加快建设“活力抚顺”。

　　深入实施强县壮区、乡村振兴战略。继续输水于乡村、放水于乡镇、蓄水于县区，大抓强县壮区，8个工业园区产值增长16％，8个服务业集聚区收入增长11％，4个农产品加工集聚区收入增长37％，17个特色产业小镇产值增长42％，8个特色小城镇产值增长12％，支持开发区、高新区率先发展，高新区尽快晋升国家级。大抓乡镇升级，深化乡镇街道财税管理体制创新，做实做深“粮头食尾”“农头工尾”文章，持续壮大主导产业，全面铺开“飞地经济”，财政收入2000万元以上乡镇升至17个，街道发展开创新局。大抓乡村振兴，精准使用涉农资金，强化科技兴农、金融惠农，加快农业产业化，深入实施“互联网＋”行动，土地规范流转比例70％以上，建设特色农产品基地20个，改造标准化养殖小区20个，村集体经济增长10％以上，多点支撑更加高效有力。

　　2.聚焦产业振兴，加快新旧动能转换。坚持壮大实体、工业立市不动摇，切实在“优老”“开原”“育新”上下硬功夫、见大成效。

　　全力优化“老字号”。始终把传统产业作为稳增长的压舱石，支持石化公司、矿业集团等骨干企业信息化智能化改造，大力运用新工艺、开发新产品，推动装备制造、冶金炼化等迈向高端化、成套化，实施技术改造项目110个、总投资460亿元，年内投资38亿元。深入实施地企大融合、企业大协作，围绕骨干龙头企业，大力发展配套产业、延伸产业，共建深加工项目30个，力争把“原字号”“初字号”就地“吃干榨净”，做到“优”出新效益、“开”出新产值。

　　全力培育“新字号”。始终把新兴产业作为奋力赶超的助推器，大胆解放思想，下好先手棋，强力推进“IT＋”产业发展，依托中国制造2025，全力争创国家5G试点城市，新引进大数据、人工智能等前沿科技企业20家。大力发展储能及新能源产业，氢燃料电池、新能源汽车等项目取得实质进展，清原抽水蓄能电站二期项目争取获批。加快发展医疗服务、现代医药、健康养老、健康养生等新兴业态，5家研发机构、12个康养项目建成达效，健康产业产值达20亿元。壮大以旅游为牵动的现代服务业，新产业新业态逐步成长为振兴发展的新动能。

　　全力实施创新驱动。始终把创新作为第一动力，大力发展高新技术产业，落实重大科技成果转化等扶持政策，将科技研发投入占地区生产总值比重提高至1.3％，转化科技成果70项，技术合同成交额6亿元，国家高新技术企业达110家、超额完成三年倍增计划。深入实施知识强企、质量兴企行动，万人发明专利拥有量增长6％，8个特色名优品牌实现产值122亿元、增长10％。健全“引育用留”人才政策，全力培育用好现有人才、广招八方英才，引进各类人才2000人以上、科研团队8个，全力推动高质量发展、建设“创新抚顺”。

　　3.聚焦项目建设，全力扩大对内对外开放。项目是扩大经济总量、增强发展后劲的核心根本，必须强力攻坚、持续发力。

　　狠抓项目建设。切实以项目论英雄、比高下，强力开展“双招双引”，组织部门、县区、乡镇齐动手，动员社会组织、中介组织和经纪人广参与，紧盯“一带一路”战略、京津冀、长三角、珠三角等产业转移，大力发扬“五皮”精神，大打招商攻坚战、歼灭战，实现全员招商、全民招商，签约亿元以上正式合同项目35个、总投资100亿元。继续强化“项目管家”制度，坚持高频调度、挂图作战、节点攻关，严格中梗阻责任倒查，坚决打通项目落地“最后一公里”，促进沈白客专、抚顺国际物流商贸城等重大项目快落地、快建设，全年开复工亿元以上重点项目110个、完成投资145亿元。

　　狠抓扩大开放。坚持开放的宽视野、大手笔，积极参与辽宁“一带一路”综合试验区建设，广泛融入中日韩经济合作、中蒙俄经济走廊建设，推动优质钢材等大宗商品多元化出口，进出口贸易总额增长7％。全力支持企业“走出去”，推动罕王印尼产业园、永茂新加坡研发中心等国际合作项目高效建设。精准定位合作的国家地区，大力推介优势项目，全力引进海外投资，实际利用外资增长10％以上，更多更好地分享开放红利。

　　狠抓先行先试。敢于祛禁锢、放手脚、夺先机，深入研究国家政策导向，超前包装对接项目，推动项目政策早承接、早见效。深入研究新兴产业发展趋势，率先布局前沿产业，创新开展化工、钢铁等商品交易平台建设。深入研究江浙等地改革创新模式，全面落实省自贸区改革创新经验，大力复制推广先进做法，确保外地能做到的事，抚顺就能做到，努力做得更好。

　　4.聚焦服务企业，打造最优营商环境。企业是社会财富的直接创造者，深化改革、优化环境一切都是为了企业更好发展。始终像珍爱眼睛一样爱护企业，我们说到做到、长期践行。

　　全力优化营商环境。勇于自我革命、破旧出新，进一步做实夯实“三集中、三到位”和“3450”改革，持续优化审批流程，深化“一网一门一次”改革，大力推行“网上审批”，“一窗受理”和网上可办率达80％以上。坚决破除“官本位”，大力倡导“商为重”，实打实包保服务企业，钉钉子解决困难问题，如实兑现政策承诺，当好服务企业“店小二”，实现“人人皆为环境”，新增市场主体2万户。扎实推进“8890”综合服务平台建设，强力推行“零打扰”行动，严厉查处曝光企业、群众“办事难”问题，全方位整治吃拿卡要等行为，努力实现在抚顺办事不求人，构建“亲”“清”政商关系。

　　全力深化各项改革。紧扣优化服务、增值增效抓改革，扎实推进供给侧结构性改革，进一步深化“放管服”改革，实现按需优质供给，持续降低企业成本。全面深化国资国企改革，推动抚顺水泥厂等尽快改制重组，加快发展混合所有制经济，全力盘活闲置资产，做强做优做大国有资本。严控政府新增债务，坚决完成年度债务化解目标。强力发展金融及类金融产业，充分利用大数据技术，搭建中小微企业金融服务平台，全力支持企业上市挂牌，投放贷款和其他融资达1000亿元，五大平台公司投融资增长10％以上，让抚顺的企业融资不再难、不再贵。

　　全力壮大民营经济。始终与民营企业同呼吸、共命运，全盘落实各项支持政策，加大减税降费奖补力度，进一步畅通与企业家沟通渠道，深入开展“解决民营企业历史遗留问题”专项行动，助力企业轻装发展。全面放宽市场准入，打造公平竞争的市场环境，推动银企、院企及企业间紧密对接合作，全力帮助企业解难题、找原料、拓市场、提效益，让企业家更愿发展更敢发展，民营企业产值增长12％。深入实施“名企名品、名家名匠”工程，依法保护企业家权益，大力表奖有突出贡献的企业家、抚顺工匠和创新创业者，让他们成为最可亲可敬的人。

　　5.聚焦绿色发展，建设美丽家园。坚持优美生态的金招牌，强力推进全方位生态环境保护治理，促进绿水青山成为金山银山。

　　强化生态保护。坚决整改环保督察反馈问题，巩固22条（段）城市黑臭水体治理成果，争创山水林田湖草国家试点，确保河流水质优良率达75％以上，大伙房水源水质保持Ⅱ类标准。协同共治大气污染，PM2.5下降至40微克每立方米，空气质量达标率不低于77.4％。扎实推进农业清洁生产，秸秆综合利用率达87％，化肥农药使用量大幅下降。继续加大松材线虫病防治力度，着力推动由疫区管控向疫点管控转变。完成人工造林、封山育林14.4万亩，森林覆盖率达68.5％，让抚顺的天更蓝、山更绿、水更清。

　　壮大绿色产业。着力推动“产业生态化、生态产业化”，大力发展“生态＋现代农业”，全面扩大食用菌、中药材等种植规模，进一步做大精深加工，打造更多名品名牌，推动绿色优质农产品走向大市场、获得高收益。大力发展“生态＋旅游”，深入实施景区景点升级工程，加快聚隆滑雪场等重点项目建设，实现冰雪旅游、冰雪运动、冰雪文化、冰雪装备一体化发展，打造四季旅游、全域旅游新格局，红色游、生态游、冰雪游、健康游等享誉全省全国，旅游总收入增长13％以上，生态效应得到最大释放。

　　提升城乡面貌。着力实施海绵城市、智慧城市建设，积极推进以城东三期为代表的产城融合发展模式，打通新城东路和贵城通道，城乡电网改造投入12.7亿元，智慧交通、智慧公交全面升级，群众出行更加便利快捷。继续还绿还美于城，维修改造棚改新区、弃管小区460万平方米，推动绿化净化进社区、入小巷，集中治理随意停车、乱建乱挖、乱扔乱堆等问题，农村垃圾分类处置实现乡镇全覆盖，做到建的精准、管的精细，加快打造一批美丽乡村、美丽社区，早日建成名副其实的“美丽抚顺”。

　　6.聚焦惠民利民，共享振兴成果。始终坚守平民情怀、为民情怀，涉及群众的事，再难也要办好，再小也要做实，切实兜住兜好民生底线。

　　强化社会保障。进一步加大民生支出，全力攻坚“3＋7”历史遗留问题，稳步推进厂办大集体改革，千方百计确保养老金按时足额发放。强力实施精准扶贫，确保4574名贫困人口全部脱贫。进一步扩大医保参保范围，完善社会救助体系，全力维护老人、妇女、儿童、残疾人等弱势群体利益，新农合筹资标准提高30元，城乡低保和特困供养标准分别提高5％。全力鼓励就业创业，为3.2万高校毕业生、农村劳动力提供就业帮扶，培训城乡劳动力1.2万人，培育创业带头人700名以上，保障群众多渠道增收，切实让群众的腰包鼓起来、生活富起来。

　　提升城市品质。持续扩大学前教育普惠范围，全面推行中小学课后服务，强化师风师德建设，进一步提高各级各类学校的教育教学质量。推进医联体基层全覆盖，大幅降低群众医疗支出。积极出台鼓励生育政策措施，稳步提高生育率。大力发展老龄事业，加快日间照料中心等建设。提高体育场所使用效率，增强全民身体素质。大力繁荣文艺创作，市民文化活动中心建成投用，工业遗迹遗产得到有效保护。扎实做好民族宗教、军地军民融合工作。大力传承和弘扬雷锋精神，不断用雷锋精神建城育人，着力建设雷锋学院，积极培育文明新风，全力打造“雷锋之城”，建设“文明抚顺”。

　　创新社会治理。坚决打好信访“减存控增”攻坚战，信访存量化解75％，新访下降60％。深化司法体制改革，完善治安防控体系，有效遏制违法犯罪，维护社会公平正义。从严从快查处涉黑涉恶犯罪及其“保护伞”，坚决铲除黑恶势力滋生土壤。严格落实安全监管责任，狠抓生产安全、食品安全、交通安全，坚决防止重特大安全事故发生，让广大群众共建共享和谐社会。

　　今年，我们还将努力办好10件惠民实事。一是解决2.4万户居民“办证难”问题，推动1195户居民顺利回迁。二是新改建农村公路110公里、“一事一议”村路238公里。三是启动城市配水管网5年改造计划，改造老旧管网45公里。四是深化便民办税春风行动，推动7类372项原国地税业务实现同城通办。五是发放“惠农贷”1亿元以上，应急贷款突破5.5亿元。六是新建全民健身场地110处。七是新建改造农村无害化厕所4500座。八是为6500名灵活就业人员发放社会保险补贴3000万元。九是推动居家养老“六助”入家庭，惠及老龄群众22万人次。十是为1.4万名困难学生发放助学金2920万元，关爱救助贫困妇女儿童3.5万人，为贫困残疾人适配基本辅具2000件。

　　7.聚焦忠诚担当，打造有为政府。抢抓机遇，加快振兴，政府必须带头担当，率先作为。

　　提高政治站位。深入学习贯彻习近平总书记在抚考察和深入推进东北振兴座谈会上的重要讲话精神，树牢“四个意识”、坚定“四个自信”、坚决做到“两个维护”，坚持党的全面领导，以自我革命的勇气和胸怀，跳出思维定势，打破条框限制，健全奖惩机制，激励全市敢为人先、奋勇争先，推动重要讲话精神落地落实。

　　强化苦干实干。时刻以振兴抚顺为己任，强化家国情怀、使命担当，带头转作风、树形象，全力以赴“重强抓”“实好干”，决不坐而论道、讲大话空话。切实从我做起、从政府班子做起，大兴艰苦奋斗、真抓实干之风，夜以继日办实事、勤政为民出实招、攻坚克难求实效，力推扁平化管理模式，说一件、干一件、成一件，全面提高工作效率，用我们的辛苦指数换取百姓的幸福指数。

　　严格依法行政。严格按照法定权限和程序行使权力，始终依法决策、依法行政。坚持新官理旧账，履诺不走样，以政府诚信带动社会诚信。虚心接受人大、政协和社会各界监督，主动回应群众关切，确保代表委员建议提案解决率稳中有升，让百姓真切感受到政府离自己很近、跟自己很亲。

　　加强廉洁自律。坚决落实全面从严治党主体责任，持续净化修复政治生态，严格执行中央“八项规定”，严格控制“三公”经费支出，强化预算执行和审计揭示问题整改，加强国有资产监管，及时堵住工作漏洞。驰而不息正风肃纪，力戒形式主义、官僚主义，坚决治理庸懒散，严惩腐败行为，做到无禁区、零容忍、全覆盖，努力实现干部清正、政府清廉、政治清明。

　　各位代表，前进号角已经吹响，战略机遇时不我待。让我们更加紧密地团结在以习近平同志为核心的党中央周围，在省委、省政府和市委的坚强领导下，团结带领全市人民，解放思想、埋头苦干、砥砺前行，以新气象、新担当、新作为，为决胜全面建成小康社会、加快全面振兴全方位振兴而努力奋斗！
